# A rare form of male pseudohermaphroditism—Persistent Mullerian Duct Syndrome

**DOI:** 10.1093/jscr/rjac596

**Published:** 2022-12-30

**Authors:** Raazia Ramzan, Naveed Ali Khan, Abdul Khalique, Munira Abdul Aziz

**Affiliations:** Dow University of Health Sciences, Karachi 74200, Pakistan; Dow University of Health Sciences, Karachi 74200, Pakistan; Dow University of Health Sciences, Karachi 74200, Pakistan; Dow University of Health Sciences, Karachi 74200, Pakistan

**Keywords:** Persistent Mullerian Duct Syndrome, bilateral undescended testes, subtotal hysterectomy and bilateral orchiectomy

## Abstract

Persistent Mullerian Duct Syndrome (PMDS) is a rare disorder of defective sexual development in males. It is characterized by aberrant synthesis or inadequate action of Mullerian inhibiting factor resulting in derivatives of Mullerian duct, i.e. uterus, fallopian tube and upper vagina, to persist in a phenotypic male with 46XY karyotype. Here, we report a 19-year-old male with bilateral undescended testes. Further evaluation revealed that the patient had both his testes placed intra-abdominally along with a rudimentary uterus.

## INTRODUCTION

Persistent Mullerian Duct Syndrome (PMDS) is an autosomal recessive disorder where a phenotypic male with normal external genitalia has a concurrent uterus, fallopian tube and/or upper third of vagina. It results from mutations resulting in the inability of Sertoli cells to produce anti-Mullerian hormone (AMH) or a defect in AMH Type II receptor, both of which are involved in male sex differentiation. PMDS is usually an incidental finding in patients with cryptorchidism. Mostly, only one testis is undescended in such patients with the other one present in its typical position; however, both testes can also be undescended.

## CASE REPORT

A 19-year-old male was admitted to the department of general surgery at a tertiary care hospital with an empty scrotum. On examination, he had a typical male appearance with well-developed secondary sexual characteristics and a normal distribution of axillary and pubic hair. His penis appeared normal and his testes were not palpable. His abdomen was soft, non-tender, and had no palpable mass or visceromegaly.

**Figure 1:**
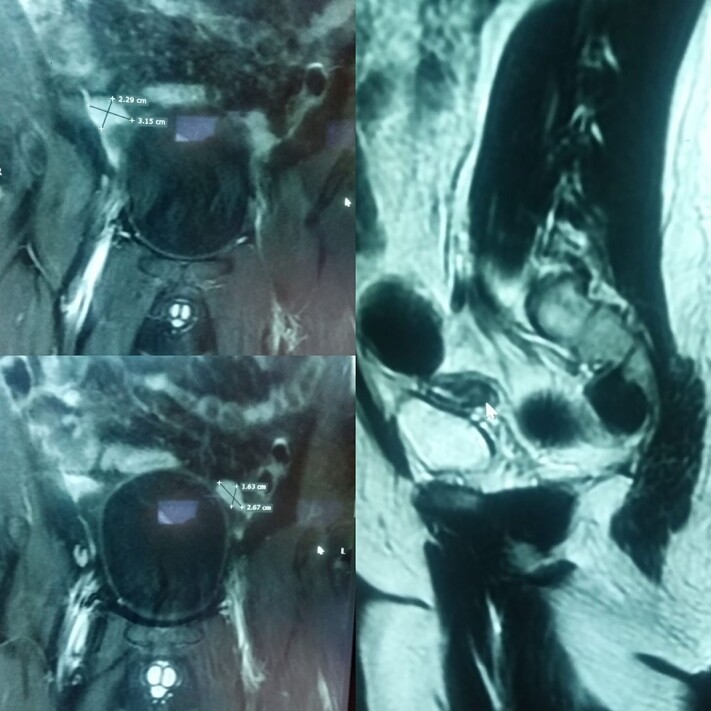
(**A**) and (**B**) are radiographic images showing well-defined enhancing areas in the lower abdomen likely to be testes. (**C**) shows the presence of a soft tissue density structure, i.e. a rudimentary uterus posterior to the urinary bladder.

His ultrasound demonstrated an empty scrotum and the testes were not detected in any ectopic location including the inguinal canal and pelvis bilaterally. Subsequently, a computed tomography (CT) scan with IV contrast was performed, which revealed well-defined enhancing areas in the lower abdomen measuring 2.5 cm × 2 cm on the left and 2.5 cm × 1.6 cm on the right correlating to the undescended testes. Furthermore, a soft tissue density structure likely to be a rudimentary uterus measuring 5.8 cm × 4 cm was seen posterior to the urinary bladder (Fig. 1).

The patient underwent semen analysis, which showed azoospermia.

His hormonal analysis showed normal values of the following:

1. Serum anti-mullerian hormone, i.e. 11.95 ng/ml.
2. Sex hormone binding globulin, i.e. 15.77 nmol/L.
3. Serum testosterone, i.e. 9.16 nmol/L.
4. Estradiol, i.e. 17.43 pg/ml.

However, he had increased

1. LH, i.e. 19.89 IU/L and
2. FSH, i.e. 23.12 IU/ml.

A tissue from buccal mucosa was sent for karyotyping, which showed a 46XY configuration of chromosomes.

**Figure 2:**
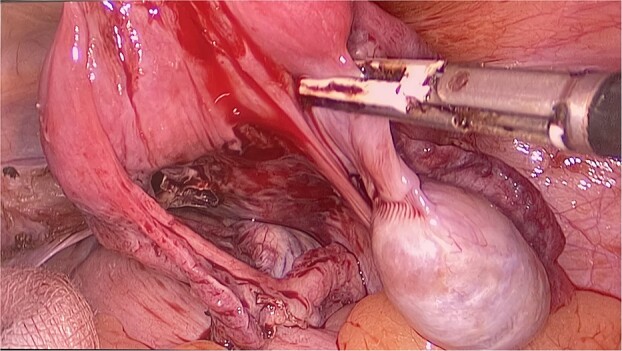
The uterus and testes were visualized in the lower abdomen by a laparoscope. Testes were seen attached to the uterus through the Infundibulopelvic ligament.

**Figure 3:**
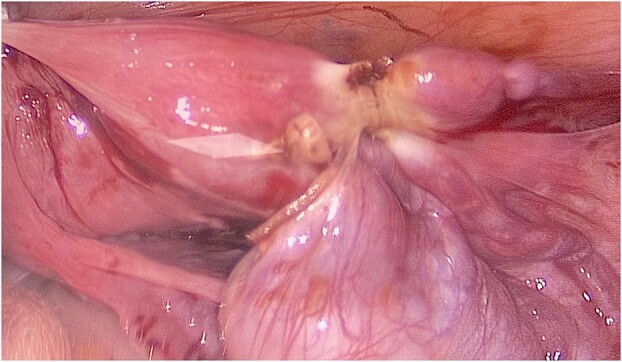
A rope-like band of connective tissue called the round ligament can be seen attaching the uterus to the abdominal wall.

**Figure 4:**
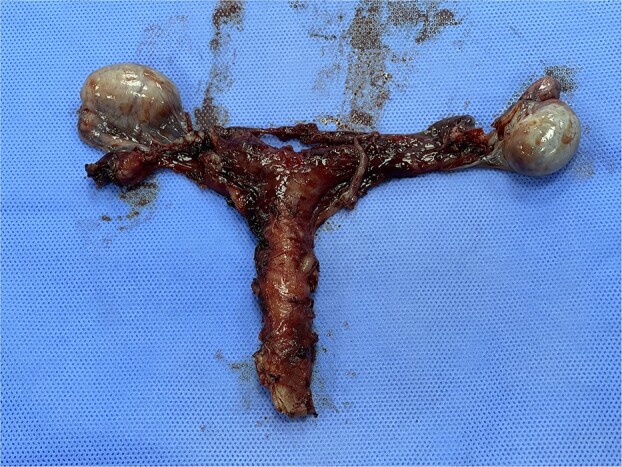
Subtotal hysterectomy and bilateral orchidopexy were performed. A tubular uterus can be seen giving off a suspensory ligament which is connecting the uterus to the testes. A coiled epididymis can also be seen attached to the testes.

The patient and his family were counseled about the patient’s situation and were informed of the available options. A well-informed consent was obtained from him and his parents for the removal of the rudimentary uterus and both his testes. We used a laparoscopic approach to visualize the presence of testes in the right and left iliac fossas, which were attached to a rudimentary uterus, located posterior to the urinary bladder through the infundibulopelvic ligament (Fig. 2). A round ligament was also seen attaching the uterus to the abdominal wall (Fig. 3). Using the same approach, the infundibulopelvic ligament was ligated followed by the dissection of the round ligament. Uterine vessels were skeletonized and ligated. After adhesiolysis, both the testes and uterus were dissected and placed in an endobag that was then retrieved through the umbilical port and sent for histopathology (Fig. 4).

The post-operative period for this patient was uneventful and, hence, he was discharged on the second post-operative day. He was also started on exogenous testosterone to maintain virilization.

Biopsy of his testes revealed benign atrophic changes with no evidence of germ cell neoplasia, granuloma or malignancy. Section of his uterus showed endometrial and endocervical lining without the presence of malignancy.

## DISCUSSION

Nilson first described PMDS in 1939 [1]. Since then, around 300 cases of PMDS have been reported [2].

Although patients with PMDS often present in their pediatric or adolescent years, the oldest age of presentation was 77 years [3]. Its presentation depends upon the anatomical variant of the disease. In the most common form, hernia uteri inguinalis, a solitary testicle is often located in the scrotal sac, whereas the uterus and fallopian tube are positioned in or near the inguinal canal. In another variant, transverse testicular ectopia, both testicles and fallopian tubes are contained within a single scrotal sac or inguinal canal. In the rarest type of PMDS, both testicles are located intra-abdominally, which accounts for <10% of all cases [4]. This patient had the rarest variety, sometimes known as the female form, because neither the scrotal sac nor the inguinal canal contained any testicles. This condition could be confused with acquired or congenital cryptorchidism, which is more common with a frequency of 1.6% in adult males. Bilateral cryptorchidism is present in 22% of all such cases [5]. Most of the time, PMDS is diagnosed incidentally or during surgery. Other times, the presenting complaint is of an empty scrotum or infertility [6]. PMDS is also associated with other conditions. A review of 157 patients found gut atresia to be the most common association, and others include hypospadias, Hirschsprung disease, horseshoe kidney, mental retardation and renal abnormalities [7].

Ultrasonography is the initial diagnostic modality of choice. It tells us about the location of testicles along its path of descent and other sexual organs, hernial sac and contents, uterine wall, etc. However, it is not very specific for the identification of intra-abdominal testicles [8]. CT scan can help identify all the structures and is superior to ultrasonography for diagnosis of intra-abdominal testicles. Magnetic resonance imaging is considered the gold standard due to its resolution, correct identification ofabdominal structures, and is superior to both ultrasonography and CT scan [9]. Serum AMH levels are also helpful in identifying the cause of PMDS [10]. Karyotyping is essential in cases of PMDS. Although most cases are karyotype 46XY, Dadheech et al. have reported a rare case of PMDS with Klinefelter syndrome [11].

Treatment strategy has remained controversial, especially regarding the removal of Mullerian remnants. There are 15–40% chances of germ cell tumors in patients with PMDS necessitating intervention [12]. This is now mostly done with laparoscopic assistance. In case of any abnormal mass detected on imaging, tumor markers are also done, and, if positive, laparotomy is preferred over laparoscopy. For children under 12 years, orchidopexy and removing all Mullerian duct structures is usually the preferred approach [13]. This brings the testicles inside the scrotum and helps maintain fertility and observe testicles for any malignancy in future. For an older age, orchiectomy is preferred [14].

## CONFLICT OF INTEREST STATEMENT

None declared.

## FINANCIAL SUPPORT AND SPONSORSHIP

None declared.

## DATA AVAILABILITY

All data used for this article is publicly available and accessible online.
